# Cooperative Dynamics of Highly Entangled Linear Polymers within the Entanglement Tube

**DOI:** 10.1021/acsmacrolett.3c00738

**Published:** 2024-03-01

**Authors:** Margarita Kruteva, Jürgen Allgaier, Michael Monkenbusch, Rustem Valiullin, Ingo Hoffmann, Dieter Richter

**Affiliations:** † Jülich Centre for Neutron Science (JCNS-1) and Institute for Biological Information Processing (IBI-8), Forschungszentrum Jülich GmbH, 52428 Jülich, Germany; ‡ Felix Bloch Institute for Solid State Physics, Leipzig University, 04103 Leipzig, Germany; § Institut Laue-Langevin (ILL), 71 avenue des Martyrs, 38000 Grenoble, France; ∥ Jülich Centre for Neutron Science (JCNS-2) and Peter Grünberg Institute (PGI-4), Forschungszentrum Jülich GmbH, 52428 Jülich, Germany

## Abstract

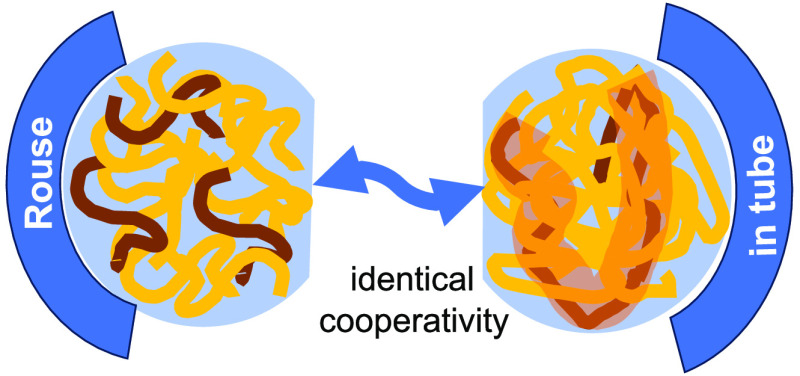

We present a quantitative comparison of the dynamic structure factors from unentangled and strongly entangled poly(butylene oxide) (PBO) melts. As expected, the low molecular weight PBO displays Rouse dynamics, however, with very significant subdiffusive center-of-mass diffusion. The spectra from high molecular weight entangled PBO can be very well described by the dynamic structure factor based on the concept of local reptation, including the Rouse dynamics within the tube and allowing for non-Gaussian corrections. Comparing quantitatively the spectra from both polymers leads to the surprising result that their spectra differ only by the contribution of classical Rouse diffusion for the low molecular weight melt. The subdiffusive component is common for both the low and high molecular weight PBO melts, indicating that in both melts the same interchain potential is active, thereby supporting the validity of the Generalized Langevin Equation approach.

High molecular weight polymers in the melt and in dense solutions interpenetrate heavily and entangle with each other. The resulting topological constraints have successfully been described in terms of the empirical tube model, where the constraints are modeled by a tube restricting lateral motion with respect to the chain profile.^1−3^ This reptation model, even though based on *ad-hoc* assumptions, has been very successful describing chain rheology as well as microscopic aspects of chain dynamics. Subsequently more fundamental ideas, such as mode coupling theory (MCT) or generalized Langevin Equation (GLE) approaches, were brought forward. In the MCT of Schweizer,^4,5^ the tube ideas are replaced by a full set of *N* (*N*: number of monomers in one chain) coupled GLE for the coupled motion of all monomers, including space-time nonlocal memory. Extending Schweizer’s approach of a one tagged chain motion, Guenza^6−8^ considered the relative or two-chain motion, where effective and explicit two-chain interactions are included via an interchain potential of mean force (PMF). As a result of chain interpenetration, the chain motions are coupled within the range of their radius of gyration *R*_g_ by a Gaussian interchain PMF. Both theories describe strong interchain couplings that generate the constraints and lead to cooperative chain dynamics.

Recently we have studied the dynamics of short chain tracers in long strongly entangled matrices for polyethylene (PE) and for poly(ethylene oxide) (PEO).^9,10^ Both experiments showed that independent of the tracer molecular weight, the motion of the tracers is significantly influenced by the interaction with host: we call this phenomenon cooperative dynamics, which is limited to the entanglement distance or tube diameter of the host. At larger distances, Fickian diffusion takes place that does not show signs of cooperative dynamics.

With this in mind, we performed an experiment on poly(butylene oxide) (PBO) melts using an unentangled short chain and a strongly entangled melt (*N*/*N*_e_ = 20, or considering the d-matrix: *N*/*N*_e_ = 24 end-to-end distances *R*_e_ = 305 Å for the large chain and 81 Å for the small one) with the aim to scrutinize the short time Rouse regime within an entangled melt and to search for signatures of cooperativity in the long chain system. The idea was to investigate a short chain melt that should be characterized by Rouse dynamics and a highly entangled melt, where based on the tracer experiments, we expected cooperative motion, which would be different to that in the short melt. Gerstl et al.^11^ gave estimates for the entanglement molecular weights *M*_e_ for PBO as 8.8 kg mol^–1^. We chose PBO, because it has a large tube diameter (about *d* = 70 Å), is rather flexible, and should display a pronounced Rouse regime in the accessible dynamic structure factor. We studied both melts at two temperatures (415 and 450 K) by Neutron Spin Echo (NSE)^12^ in a time window of about 500 ns. Using the measured Rouse rates *Wl*4, the entanglement times τ_e_ = *d*^4^/(*Wl*4 π^2^) with *d* = 70 Å come out as 295 ns for 415 K and 147 ns at 450 K well within the time window of the NSE experiment.^11^

The following results stand out: (i) Independent of any modeling, the spectra from the highly entangled PBO200 K and the unentangled PBO12K melts only differ by the Rouse diffusion contribution to the PBO12K spectra. (ii) The subdiffusive, cooperative center-of-mass motion in the Rouse regime shows itself also in segmental or internal motion within the tube constraints. (iii) Thus, the dynamics of highly entangled chains in the cross over regime from Rouse to local reptation shows the same interaction effects that also act within the low *M*_w_ melt. (iv) These results strongly support the GLE approach postulating the same PMF for unentangled and entangled chains and further limiting the cooperativity by the size of the tube constraints.

The PBO polymer were synthesized by anionic polymerization using a procedure presented elsewhere^13^ (see also Supporting Information). The synthesis of deuterated butylene oxide (dBO) is described in ref (14).^14^ The achieved polymers, which were characterized by a combination of GPC and light scattering, are listed in Table S1 (see SI).

The samples each containing 10% hydrogenous and 90% deuterated polymer were obtained by dissolving the corresponding amounts of polymers in toluene and subsequent freeze-drying. Such obtained materials were filled into flat Niobium containers of 2 mm thickness and placed perpendicular to the beam on the spectrometer.

The NSE experiments were performed at the spectrometer IN15 at the Institute Laue-Langevin (ILL).^12^ Employing neutron wavelengths of λ = 10 and 13.5 Å we studied the two h/d-PBO blends at temperatures of 415 and 450 K covering the time range 0.1 < *t* < 500 ns at momentum transfers, 0.041 ≤ *Q* ≤ 0.163 Å^–1^.^15^

Let us first look at the PBO200 K sample. It was evaluated in terms of the dynamic structure factor for reptation by Monkenbusch et al. that includes Rouse behavior at short times, local reptation at longer times, and following the work of Guenza,^8^ allows for non-Gaussian dynamics.^16^ The important parameters are the length of an entanglement strand *N*_e_, which also determines the size of the Rouse blob within the tube; the characteristic Rouse rate  with *l*_seg_^2^ = 3C_∞_*l*_0_^2^ = 38.4 Å^2^ (C_∞_ = 5.4), *k*_B_ the Boltzmann factor; ζ_0_ the monomeric friction coefficient. The non-Gaussian (NG) corrections were described by the approach of Guenza,^8^ which we also used in our previous works.^9,16^ α_0_ is the strength of the NG-correction; *t*_max_ is the time where the NG-correction assumes its maximum value, and *t*_w_ is the width of the NG function (for details of the structure factor see SI)

A very good joint fit of the spectra from both temperatures on the time scale 0.1–500 ns was obtained (Figure 1). In this fit, we jointly varied *N*_e_ and *t*_w_. The tube was taken as symmetric. The ratios of the Rouse rate *Wl*4 and *t*_max_ are very close and inverse to each other (Table 1). Fitting revealed α_0_ = 0.1. As seen, the fit provides an excellent description of the data. We note that because the data originate from the crossover regime between Rouse and local reptation, the fit is only weakly affected by the actual size of *N*_e_: values above *N*_e_ = 180 lead to a good fit.

**Table 1:** Parameters of the Joint Fit of the PBO200 K Sample at 415 and 450 K^a^

*T* (K)	*N*_e_	*Wl*4 (Å^4^ ns^–1^)	α_0_	*t*_max_ (ns)	*t*_w_ (ns)	χ^2^
415	≥180	8506 ± 56	0.100 ± 0.002	474 ± 43	2.35 ± 0.1	4.4
450	coupled	16495 ± 146	coupled	252 ± 25	coupled	4.4

a *N*_e_ is the length of an entanglement strand; *Wl*4 is the characteristic Rouse rate; α_0_ is the strength of the NG-correction; *t*_max_ is the time, where the NG-correction assumes its maximum value; *t*_w_ is the width of the NG function; χ^2^ is the mean-squared error.

**Figure 1:**
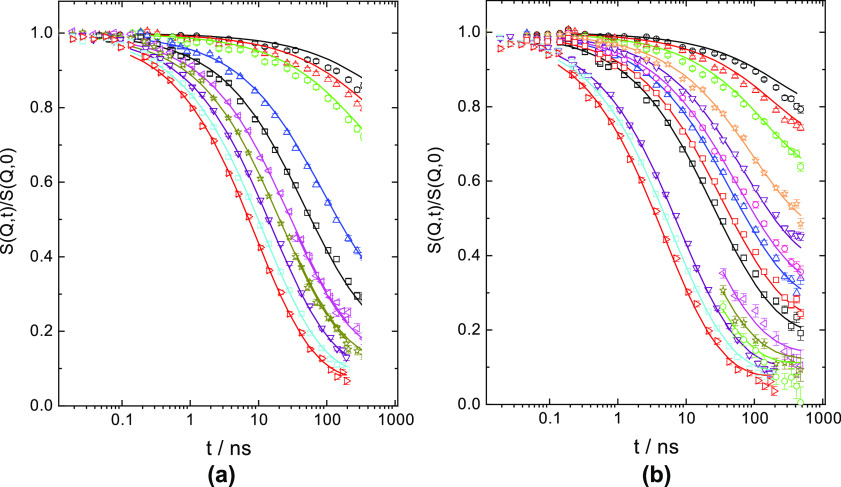
PBO200 K joint fit (a) at 415 K. *Q* values from above: 0.041, 0.056, 0.089, 0.097, 0.105, 0.120, 0.130, 0.141, 0.152, and 0.163 Å^–1^ and (b) 450 K. *Q* values from above: 0.041, 0.048, 0.056, 0.069, 0.077, 0.084, 0.089, 0.097, 0.105, 0.121, 0.129, 0.137, 0.142, 0.152, 0.163 Å^–1^.

In a next step, we compare the spectra from the PBO12K and PBO200 K samples. Figure 2 displays NSE spectra taken from the PBO12K and the PBO200 K samples at 415 K. While at short times in the initial Rouse regime both sets of spectra agree well, at longer times the PBO12K spectra decay significantly faster–they are not subject of entanglement constraints and display significant contributions from translational diffusion. At both temperatures the ratios of the spectra from the low and high molecular weight samples are virtually perfectly fitted by a single exponential *S*(*Q*,*t*)_PBO12K_/*S*(*Q*,*t*)_PBO200K_ = exp[−*Q*^2^*D*_com_*t*], suggesting that the data may be interpreted as resulting from translational center-of-mass (com) diffusion on the time scale of some hundreds of ns.

**Figure 2:**
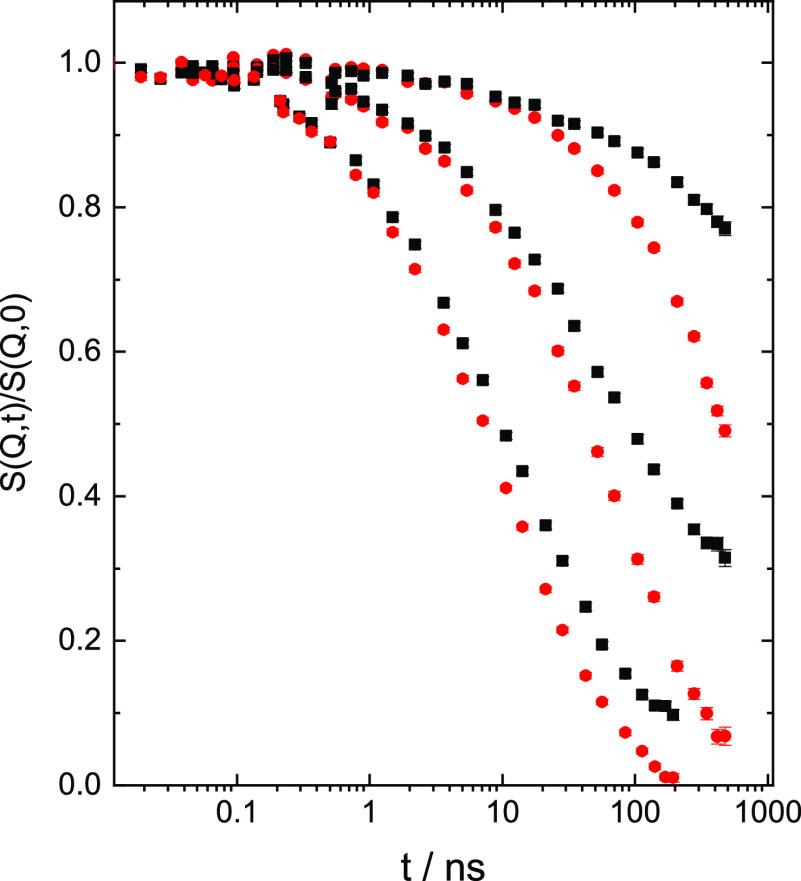
Comparison of spectra from PBO12K (red circles) and PBO200 K (black squares) at 415 K. *Q* values from above: 0.048, 0.097, and 0.152 Å^–1^.

Figure 3 presents the data in terms of com displacement:
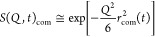
1
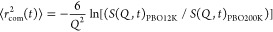
2

**Figure 3:**
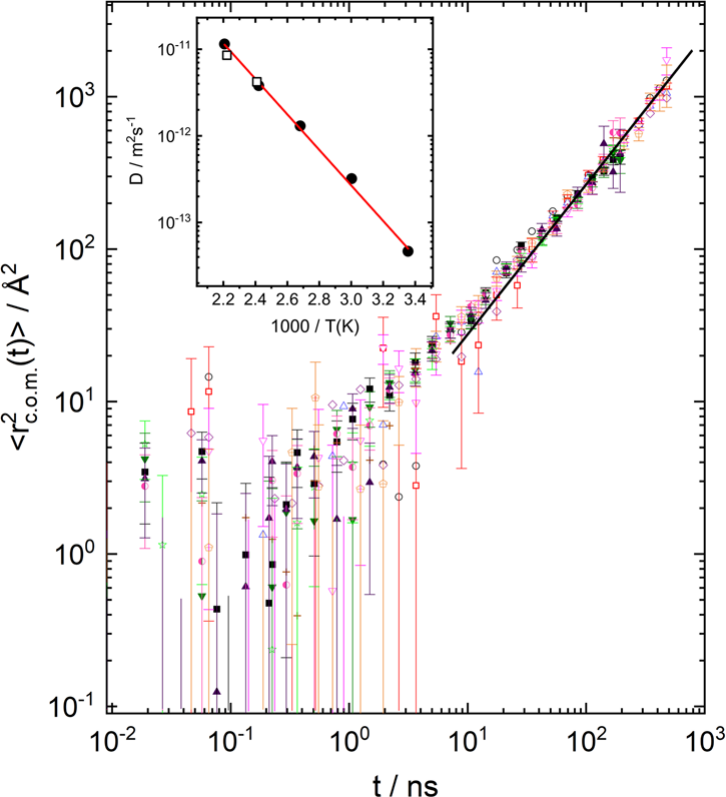
Ratio PBO12K/PBO200 K at 415 K interpreted as resulting from com displacements (*Q*-range: 0.041 ≤ *Q* ≤ 0.163 Å^–1^. Inset: com diffusion PBO12K at 415 K; circles: PFG-NMR; squares: NSE results, see text.

As may be seen, all data originating from the *Q*-range 0.041 ≤ *Q* ≤ 0.163 Å^–1^ collapse on a master curve relating to ⟨*r*_com_^2^(*t*)⟩ = 2.52*t* [Å^2^]. Similarly, the data at 450 K follow a master curve relating to a center of mass displacement of: ⟨*r*_com_^2^⟩ = 5.1*t* [Å^2^].

The corresponding com diffusion coefficients at 415 K are 0.42 Å^2^/ns and at 450 K: 0.85 Å^2^/ns. Like the ratio of the Rouse rates at 450 and 415 K the corresponding ratio of the diffusion coefficients with good precision equals to 2. Now we scrutinize the relation of the com Fickian diffusion coefficients with the related Rouse rates . For *T* = 415 K: *D*_Rouse_(415 K) = 0.44 Å^2^/ns; for 450 K *D*_Rouse_(450 K) = 0.86 Å^2^/ns in nearly perfect agreement with the values found above. Thus, the diffusion coefficients obtained by dividing the PBO12K spectra by those from PBO200 K are fully consistent with the Rouse picture for the long diffusion coefficient. The inset in Figure 3 compares long-range diffusion coefficients obtained by PFG-NMR that operates at the scale of μm and ms with the above neutron results. As may be seen, the PFG-NMR diffusion coefficients are in very close agreement with the NSE-derived values for Fickian diffusion.

To check for consistency and to find out about the subdiffusive contribution to the dynamics of the PBO12K chains, we fixed the Fickian diffusion to the values obtained from the procedures above and the *Wl*4 parameters to those from the Rouse blobs in the long-chain dynamics. As Figure 4 displays, performing a joint fit of the spectra from both temperatures, excellent results are achieved.

**Figure 4:**
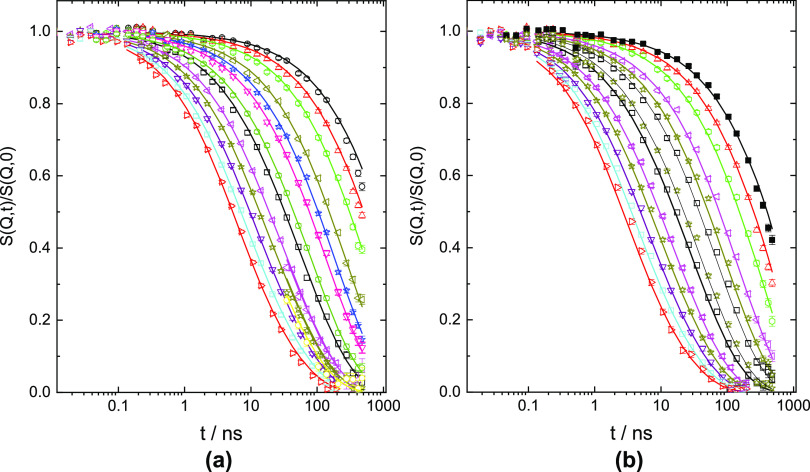
PBO12K (a) at 415 K,*Q*-values from above: 0.041, 0.048, 0.056, 0.069, 0.077, 0.084, 0.097, 0.105, 0.12, 0.13, 0.142, 0.152, and 0.163 Å^–1^. Solid lines: fit with the Rouse model including sublinear diffusion (see text, *Wl*4 = 8506 Å^4^/ns; *D*_Rouse_ = 0.42 Å^2^/ns); (b) at 450 K. Solid lines: fit with the Rouse model including sublinear diffusion (see text, *Wl*4 = 16495 Å^4^/ns; *D*_Rouse_ = 0.85 Å^2^/ns); *Q* values as in (a).

For both the high and low molecular weight PBO, we considered NG effects. Coupling the corresponding parameters for both temperatures, we found for the strength of the NG correction α_0_ = 0.30 ± 0.004; for *t*_max_, the fit yielded 71 ± 4 ns at 450 K and 142 ± 8 ns at 415 K. As it seems the NG-correction appears to be more significant compared to the high *M*_w_ melt. However, we note that the data are also well described, fixing the NG-correction to the result from PBO200 K and allowing for a suppression of the first Rouse mode (see SI).^17^

The cross over from Fickian to subdiffusion was found to take place at an MSD of ⟨*r*_0_^2^⟩ = 2850 Å^2^, which lies in between *R*_g_^2^ and *R*_e_^2^ (1070 Å^2^ < 2850 Å^2^ < 6400 Å^2^). The significant subdiffusive component to the spectra displays an exponent of 0.659 ± 0.002. Figure 5 shows the contribution of com diffusion to the PBO12K spectra at 415 K. As may be seen at the small *Q* = 0.048 Å^–1^, the spectrum is entirely described by com diffusion, while at larger *Q* the internal modes contribute more and more. The inset in Figure 5 shows the time-dependent com displacements. The blue dashed line depicts the Rouse part, while the black solid line includes the subdiffusive part, which at short times dominates.

**Figure 5:**
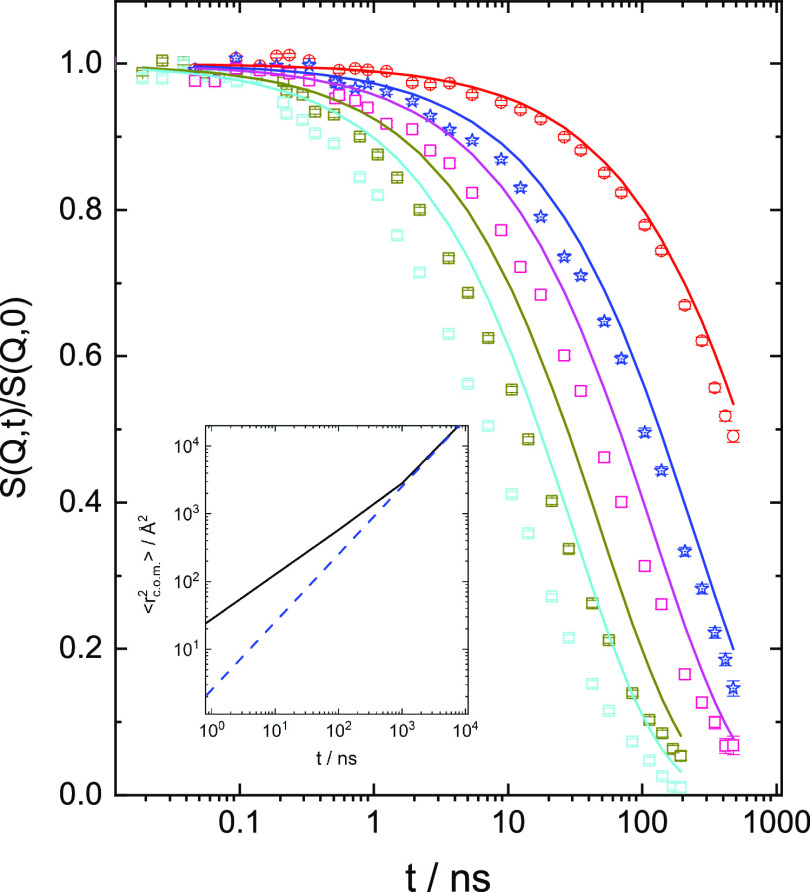
Contribution of cubic-om diffusion to the spectra from PBO12K at 415 K. *Q*-values from above: 0.048, 0.077, 0.097, 0.130, and 0.152 Å^–1^. Inset presents the com diffusion: blue dashed line is Rouse diffusion; black solid line is the com diffusion, including both Rouse diffusion and subdiffusion.

In our experiments, we have quantitatively compared the dynamic structure factors from unentangled and strongly entangled polymer melts. As expected, the low molecular weight PBO displays Rouse dynamics with an additional very significant subdiffusive component of the com diffusion. Note that at lowest *Q* (Figure 5) the dynamics is entirely determined by com diffusion, allowing for an experimentally accurate distinction from internal modes. Combining the PFG-NMR results with the NSE data, Fickian diffusion is observed from about 10–10^4^ Å. The high molecular weight strongly entangled PBO can be very well fitted with the dynamic structure factor based on the concept of local reptation including the Rouse dynamics within the tube and allowing for NG – corrections.^16^ Comparing quantitatively the spectra from both polymers led to the very surprising result that the spectra from both polymers distinguish each other only by the contribution of classical Rouse diffusion. Thereby, the proper diffusion coefficients coincide with the values calculated from the relaxation rates obtained from the short-time decay in the high *M*_w_ melt. These diffusion coefficients also agree with those from PFG-NMR measured on the mesoscopic μm scales. The subdiffusive component is common for both the low and high molecular weight PBO samples even though the data from the entangled high *M*_w_ melt result from the crossover regime from Rouse to local reptation. We emphasize that this result is independent from any modeling and arises from a direct unbiased comparison of the spectra from both polymers.

From a measurement of the com diffusion of short chain PE melts covering a chain length regime crossing the entanglement threshold, Zamponi et al. found a subdiffusive regime that became more pronounced with increasing chain length.^18^ These data could be well interpreted by Ansatz of a generalized Langevin equation for cooperative dynamics (CDGLE). The CDGLE approach relates the anomalous diffusion to the presence of an interchain potential between sections of pairs of distinct polymer chains, which correlates with the dynamics of slowly diffusing chain molecules. CDGLE was able to quantitatively account for the NSE results.^19^ Relating to this observation, it is highly likely that the subdiffusive com diffusion within the PBO12K melt originates from the same phenomenon.

In 2014, Guenza extended the CDGLE concept to strongly entangled polymer melts, postulating that cooperative dynamics takes place mainly within the tube constraints. Thereby, the interchain potential is the same for entangled chains as that for unentangled chains. However, the cooperative monomer fluctuations are confined to the entanglement volume. These theoretical predictions were very nicely corroborated by recent diffusion experiments of short tracers in highly entangled PE and PEO melts.^9,10^

Following Guenza’s approach, the unexpected results for the PBO samples may be understood. We reiterate that aside from translational com Rouse (Fickian) diffusion of the spectral shape from both samples agrees quantitatively. Thus, the cooperative motion that gives rise to com subdiffusion in the unentangled short chain melt must also take place within the tube constraints of the long chain melt; the subdiffusive part in the com diffusion is not only present in the PBO12K data but also shows itself in the long chain PBO200K dynamic structure factor. Again, we note that the spectra from the entangled chains originate from a time regime where topological constraints become important. As for the short chains, where the early time subdiffusive part is related to an interchain potential that couples the com motion of several chains,^20^ a very similar phenomenon appears to take for the chain dynamics within the tube confinement. Also, there interchain interactions between the entanglement strands give rise to cooperative motion expressed by the same type of dynamic correlations as for the short chains. The observation agrees well with the CDGLE approach assuming the same interchain potential for unentangled and entangled chains and in addition limiting the cooperative monomer fluctuation to inside the tube constraints.

Also, the outcome of our tracer experiments on short tracers^9,10^ (PEO and PE) in a strongly entangled melt supports this conclusion. These experiments also demonstrated that cooperativity is limited to inside tube constraints.

Still, it is highly astonishing that the long-time (Fickian) diffusion of the mutually interacting short chains exactly matches the plain Rouse model prediction. This undeniable observation warrants further experimental scrutiny and theoretical explanation.

## Supporting Information Available

Synthesis of polymers; Calculations of dynamic structure factor for a strongly entangled chain; Alternative description of PBO12K; Role of non-Gaussian correction for long chains; Details of the PFG-NMR experiment (PDF)
