# Supplementary material for: Cooperative Dynamics of Highly Entangled Linear Polymers within the Entanglement Tube
Source: ACS Macro Lett. 2024 Mar 1;13(3):335–40. doi: 10.1021/acsmacrolett.3c00738 (PMC10956489; doi:10.1021/acsmacrolett.3c00738)
Supplement: Supplementary file 1 — mz3c00738_si_001.pdf [file mz3c00738_si_001.pdf]

Supporting Information:

Cooperative dynamics of highly entangled linear polymers within the entanglement tube

Margarita Kruteva[1*], Jürgen Allgaier[1], Michael Monkenbusch[1], Rustem Valiullin[2], Ingo Hoffmann[3] and Dieter Richter[4]

[1]*Jülich Centre for Neutron Science (JCNS-1) and Institute for Biological Information Processing (IBI-8), Forschungszentrum Jülich GmbH, 52428 Jülich, Germany*

[2]*Felix Bloch Institute for Solid State Physics, Leipzig University, Leipzig, 04103 Germany*

[3]*Institut Laue-Langevin (ILL), 71 avenue des Martyrs, 38000 Grenoble, France*

[4]*Jülich Centre for Neutron Science (JCNS-2) and Peter Grünberg Institute (PGI-4), Forschungszentrum Jülich GmbH, 52428 Jülich, Germany*

*Corresponding author: m.kruteva@fz-juelich.de

Synthesis

General procedures. All manipulations were carried out at a high vacuum line or in a glove box, filled with argon (M Braun, Unilab). The water and oxygen levels in the glove box were usually below 1 ppm. The flasks for the manipulations were equipped with Teflon stopcocks that allowed transferring materials between the vacuum line and the glove box without contamination with air. The flasks which were exposed to overpressure were pressure tested to 12 bars. The polymers were synthesized according to a procedure already published [1]. The synthesis of deuterated 1,2-butylene oxide (dBO) is described in Ref.2 [2]. For the synthesis of the high molecular weight polymers, a solution of the initiator potassium *tert*-butanolate (KO*t*-Bu) and 18-crown-6 (18C6) was produced by dissolving 28.9 mg of KO*t*-Bu and 350.5 mg of 18C6 in 12.954 g of dry toluene.

Synthesis of h-PBO12k. The polymerization reaction was carried out using 213 mg (1.90 mmol) of KO*t*-Bu, 496 mg (1.88 mmol) of 18C6, 29.93 g of dry BO and 31 g of dry toluene at -20 °C for one day. The polymerization was terminated with 1 mL of acetic acid, all volatile material was distilled off under reduced pressure and the residue was washed 3 times with 200 mL of methanol. The product was dried under stirring at high vacuum conditions overnight and a colorless viscos liquid was obtained.

Synthesis of d-PBO12k. The polymerization reaction was carried out using 133 mg (1.19 mmol) of KO*t*-Bu, 296 mg (1.12 mmol) of 18C6, 21.05 g of dry dBO and 20 g of dry toluene at -15 °C for 3 days and -10 °C for another 4 days. The purification procedure was similar to the one described for h-PBO12k.

Synthesis of h-PBO200k. The polymerization reaction was carried out using 2.940 g of the of KO*t*-Bu/18C6 solution (0.058 mmol KO*t*-Bu, 0.293 mmol 18C6), 19.33 g of dry BO and 20 g of

dry toluene at -30 °C for 22 days. After termination with 0.5 mL of acetic acid, the polymer was precipitated in 700 mL of methanol, washed with methanol and dried under vacuum conditions for 1 h. Due to the presence of low molecular weight product, the polymer was fractionated by dissolving it in 1.25 L of toluene and adding 2.1 L of methanol, which contained 0.5 g of Butylhydroxytoluol (BHT). The turbid mixture became clear upon warming up to 31 °C. After slow cooling to room temperature overnight in a separating funnel, the lower phase was isolated, precipitated in 600 mL of methanol, washed with methanol and dried under high vacuum conditions for 2 days. 7.83 g of rubbery product was obtained.

Synthesis of d-PBO200k. The polymerization reaction was carried out using 2.220 g of the KO*t*-Bu/18C6 solution (0.044 mmol KO*t*-Bu, 0.224 mmol 18C6), 16.09 g of dry dBO and 11.4 g of dry toluene at -30 °C for 22 days. After termination with 0.3 mL of acetic acid, the polymer was precipitated in 700 mL of methanol, washed with methanol and dried under high vacuum conditions for 2 days. 11.06 g of rubbery product was obtained.

	M_n / kg mol^{-1}	M_w/M_n	$Z = M_w/M_e$
h-PBO12K	12.1	1.03	< 2
d-PBO12K	11.5	1.05	< 2
h-PBO200K	175.0	1.02	20
d-PBO200K	243.0	1.02	24

Table S1 Poly(butylene oxide) polymers and their physical characteristics: molecular weights M_n, molecular weight distribution M_w/M_n and number of entanglement per polymer chain $Z = M_w/M_e$.

Dynamic structure factor for a strongly entangled chain

Recently we achieved a more complete description of a chain undergoing local reptation within the tube model by DeGennes and Doi and Edwards [3]. There the tube is defined by a characteristic length scale (tube diameter) *d* that is also taken as its step-length, and by its contour length *L*. Refinements of the model pertain contour length fluctuations (CLF) that gradually relax tube constraints from its ends as well as the effect of constraint release (CR). The new expression emerged from the original DeGennes approximation for the dynamic structure factor. We describe the single chain dynamic structure factor in multiplying the coherent scattering functions for local reptation and Rouse motion within a Rouse blob that fills the tube laterally. The relative contribution of local reptation type fluctuations was fixed according to results obtained from the statistics of Gaussian random paths. Furthermore, including Non-Gaussian (NG) effects significantly improved the matching of experiment and model. Thus, the dynamic structure factor is refined in three steps:

Step 1: The structure factor for local reptation by DeGennes (Eq. 3.11 in Ref. 4 [4]) is fully integrated yielding two contributions to $S(Q,t)$. The elastic part is described by the Debye function of the chain with a step-length given by the tube diameter. The dynamic part assumes the form:

$$S_{locrep}(Q,t) = \frac{2N_e}{3}\sqrt{\frac{W*t}{\pi}}\left[exp\left(-\frac{l_{seg}^2 Q^2 N}{6} - \frac{N^2}{Wt}\right) - 1\right] + N_e\left[\frac{N}{3} + \frac{l_{seg}^2 Q^2 Wt}{9}\right] * exp\left(\frac{l_{seg}^4 Q^4 Wt}{36}\right) * \left\{erfc\left(\frac{l_{seg}^2 Q^2 \sqrt{Wt}}{6}\right) - erfc\left(\frac{3N + l_{seg}^2 Q^2 \sqrt{Wt}}{6\sqrt{Wt}}\right)\right\} \quad \text{(S1)}$$

With N_e the number of monomers in an entanglement strand, l_{seg} the segment length, which is taken as the monomer length, W the elementary Rouse relaxation rate, Q the momentum transfer, N the total number of monomers of the chain and erfc the complementary error function. The quantitative analysis of the random path statistics gives the relative contribution of the fluctuations to the structure factor of $\Xi = 0.41$, somewhat larger than DeGennes' value of 1/3.

Step 2: The Rouse motion within the tube constraints is treated as a Rouse blob of a size assumed to be the lateral tube dimension. The dynamic structure factor of the Rouse blob has the form:

$$S_{Rouse}(Q,t) = \frac{1}{N_{blob}}\sum_{i,j}^{N_{blob}} exp\left[\left(\frac{Q^2}{6}\right)\langle[r_i(t) - r_j(0)]^2\rangle\right] \quad \text{(S2)}$$

With

$$\langle[r_i(t) - r_j(0)]^2\rangle = |i-j| l_{seg}^2 + \frac{4R_e^2}{\pi^2}\sum_{p=1}^{N_{blob}} \frac{1}{p^2} cos\left(\frac{p\pi j}{N_{blob}}\right) cos\left(\frac{p\pi i}{N_{blob}}\right)\left[1 - exp(t\Gamma_p)\right] \quad \text{(S3)}$$

Where $\Gamma_p = 2W\left[1 - cos\left(\frac{p\pi}{N_{blob}}\right)\right] \cong \frac{p^2}{\tau_R}$ with the Rouse time $\tau_R = \tau_e = R_e^4/\pi^2 W l_{seg}^4$ and R_e being the size of the Rouse blob in terms of its end to end distance. The full dynamic structure factor including local reptation and local Rouse dynamics is then approximated by a convolution of the correlation functions in real space and correspondingly by a product in (Q,t)-space.

Step 3: Following results by Guenza, who realized the importance of NG effects, NG corrections in terms of a non-Gaussian parameter $\alpha(t)$ are included. Then, the scattering function becomes:

$$S(Q,t) \cong \frac{1}{N_{blob}}\sum_{i,j}^{N_{blob}} exp\left[\left(\frac{Q^2}{6}\right) f(Q^2)\langle[r_i(t) - r_j(0)]^2\rangle\right] \quad \text{(S4)}$$

With

$$f(Q^2) = 1 - \frac{Q^2\alpha(t)\langle[r_m(t) - r_m(0)]^2\rangle}{12} + O(Q^4) \quad \text{(S5)}$$

For a Rouse chain we have

$$\langle[r_m(t) - r_m(0)]^2\rangle = \frac{1}{N_{blob}} \frac{4N_{blob} l_{seg}^2}{\pi^2} \sum_{n,p=1}^{N_{blob}} \frac{1}{p^2} cos\left(\frac{p\pi m}{N_{blob}}\right)^2 \left[1 - exp\left\{-2W\left(1 - cos\left(\frac{p\pi}{N_{blob}}\right)\right)t\right\}\right] \quad (S6)$$

Finally, again following the results of Guenza for the functional form of $\alpha(t)$ a logarithmic Gaussian function is used

$$\alpha(t) = \alpha_0 exp[-\{[ln(t) - ln(t_{max})]^2/[2t_w^2]\}] \quad (S7)$$

With $\alpha_{max} = \alpha_0$ and t_w being the width of the distribution.

Alternative description PBO12K

The second approach to quantitatively describe the PBO12K spectra assumes that the NG-correction follows that of the PBO200K melt. Thus, in the fitting procedure we fix the NG-parameters to the values obtained for PBO200K: α_0= 0.1; t_{max}(415K) = 474 ns, t_{max} (450K) = 252 ns; t_w = 2.35 ns) and allow for a modification of the Rouse mode amplitudes for the first Rouse modes. This procedure follows the very successful description of the dynamic structure factor for polyethylene-oxide in the cross-over regime between Rouse dynamics and local reptation[5]. Table S1 presents the obtained parameters. Fig. S1 compares the experimental spectra for the PBO12K melts at 415K and 450K with the achieved dynamic structure factor.

Table S2: Parameters from the joint fit of the PBO12K dynamic structure factors.

T / K	Cross-over $\langle r_0^2\rangle$ [Å^2]	Exponent sub-diffusion	Suppressed mode	α_0	t_{max} [ns]	t_w
415	3087$\pm$28	0.66$\pm$0.001	1	0.1	474	2.35
450	coupled	coupled	1	0.1	252	2.35

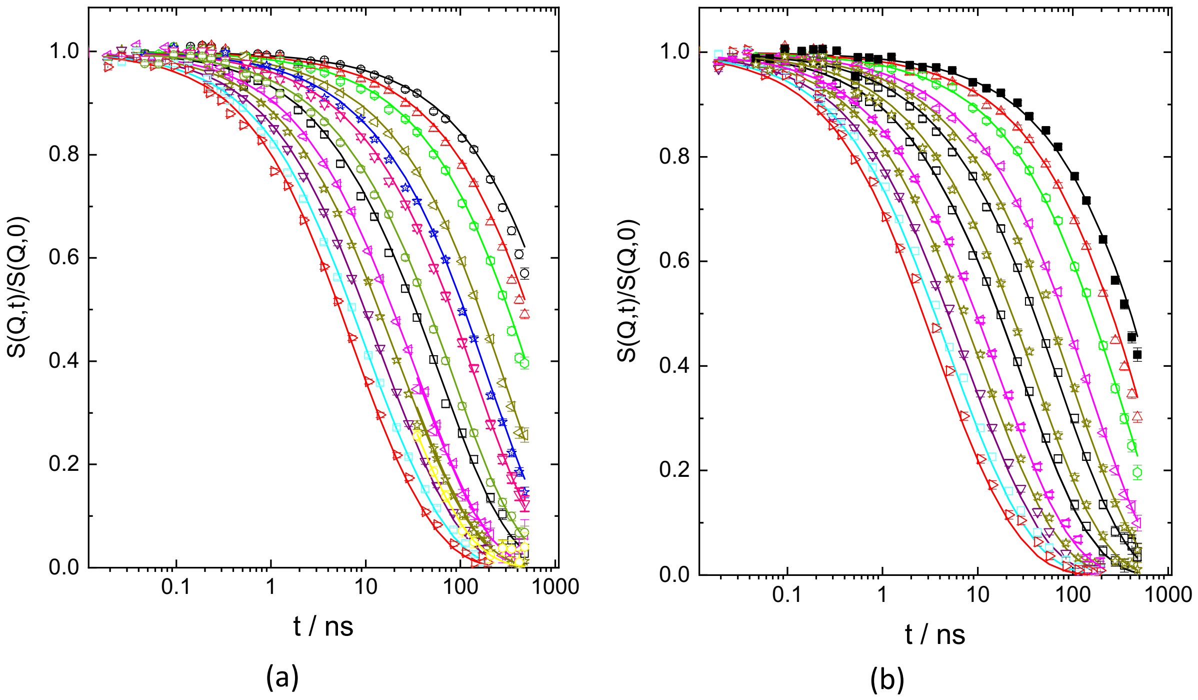

Fig. S1: PBO12K at (a) 415K and (b) 450K described in terms of mode suppression and NG-parameters from PBO200K.

Non-Gaussian (NG) correction

Figure S2 compares best joint fits of PBO200K at 415K and 450K with and without NG-corrections. Even though in general a value of α_0 in the order of 0.1 is considered as insignificant, the fit quality importantly increases if NG–corrections are considered. In particular the long time dynamics within the structure factor is modelled significantly better – an observation that holds for both temperatures.

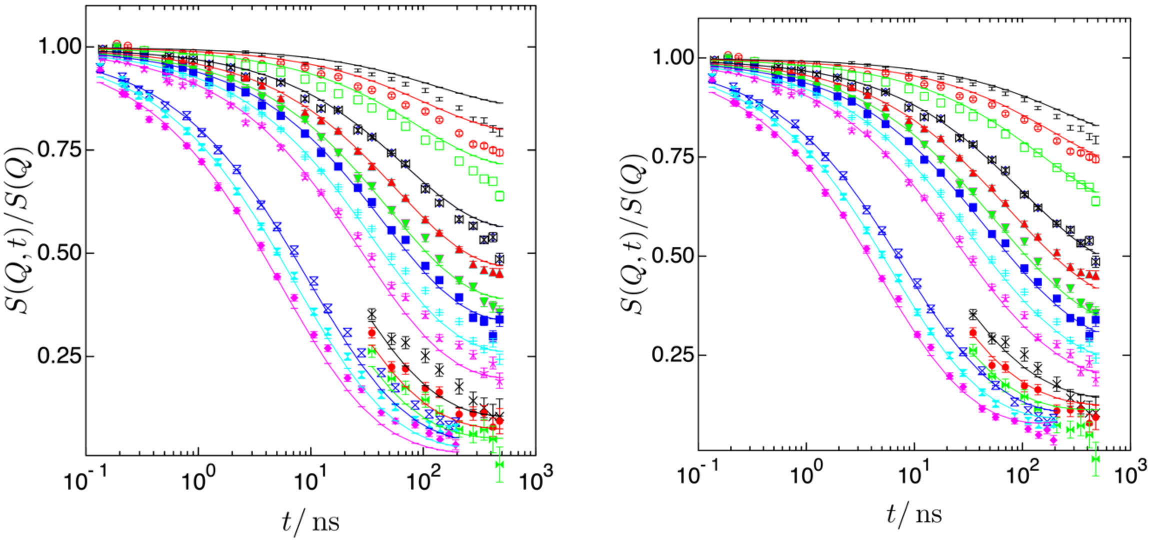

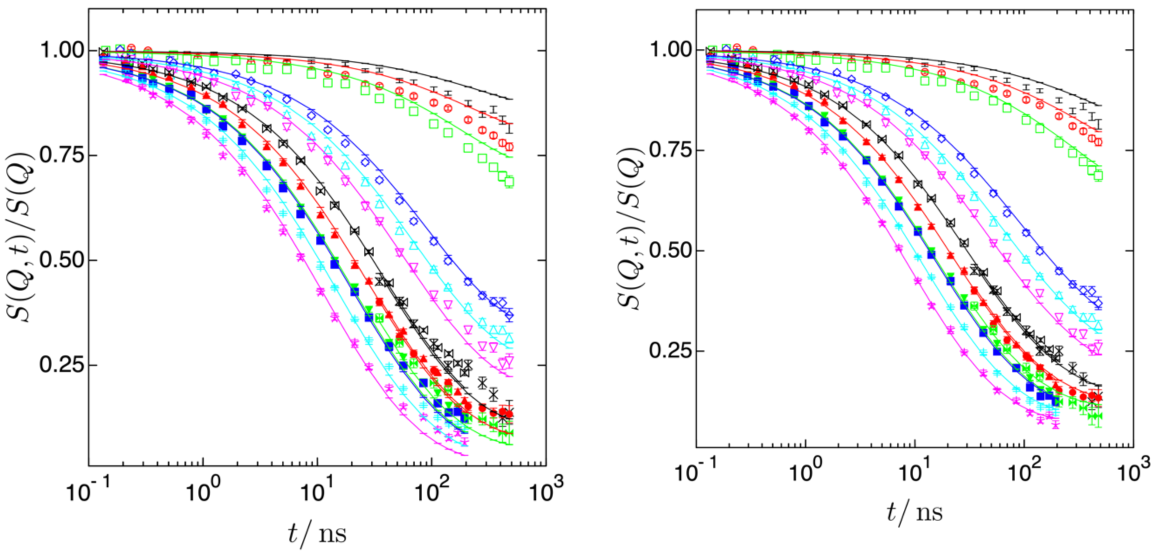

Fig. S2: PBO200K upper (450K) and lower (415K) left: best fit without NG-correction $\chi^2 \cong 10$; right side the same data employing NG – correction that yield alpha =0.1 ($\chi^2 \cong 4.5$)

PFG NMR

The diffusion NMR experiments have been performed on a home-built NMR spectrometer operating at 100 MHz proton resonance frequencies. A specially designed NMR probe-head allowed for applying high-intensity gradient pulses up to $g = 30$ T/m with extremely short gradient pulse rise and fall times in a broad temperature range from -100°C to 300°C. For the measurements the 13-interval pulse sequence shown in Figure S3 was used. For the measurements, the time interval between 90° and 180° was $\tau = 2$ ms and the gradient pulse duration $\delta = 500$ μs were used. The time interval Δ was fixed to a value yielding the diffusion time $t_d = \Delta + \frac{3}{2}\tau - \frac{1}{6}\delta = 50$ ms.

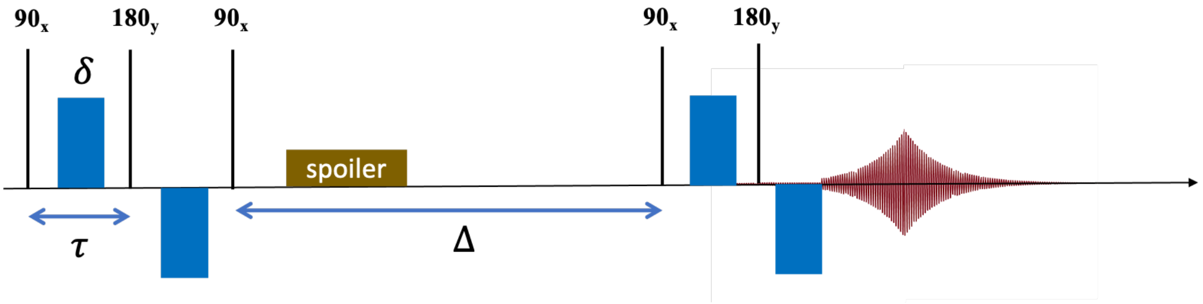

Figure S3. 13-interval PFG NMR pulse sequence for diffusion measurements.

The spin echo amplitude measured with the 13-interval pulse sequence is given by

$$S(q, t_d) = S(q, 0) \exp\left\{-\frac{1}{2} q^2 \langle z^2(t_d) \rangle\right\} \tag{S8}$$

where the wave-number q is defined as $q = \gamma\delta g$ and γ is the gyromagnetic ratio for protons. Assuming normal diffusion, the self-diffusion rate D can be related to the mean-square-displacements as

$$\langle z^2(t_d)\rangle = 2Dt_d \tag{S9}$$

yielding

$$S(q,t_d) = S(q,0)\exp\{-q^2Dt_d\} \tag{S10}$$

The experimentally measured spin-echo diffusion attenuation functions are shown in Figure S4 for different temperatures. Notably, they are of the mono-exponential shape pointing out a single diffusion rate in the system and the normal process of diffusion. The diffusion rates D reported in the manuscript were found by fitting Eq. S10 to the experimental data.

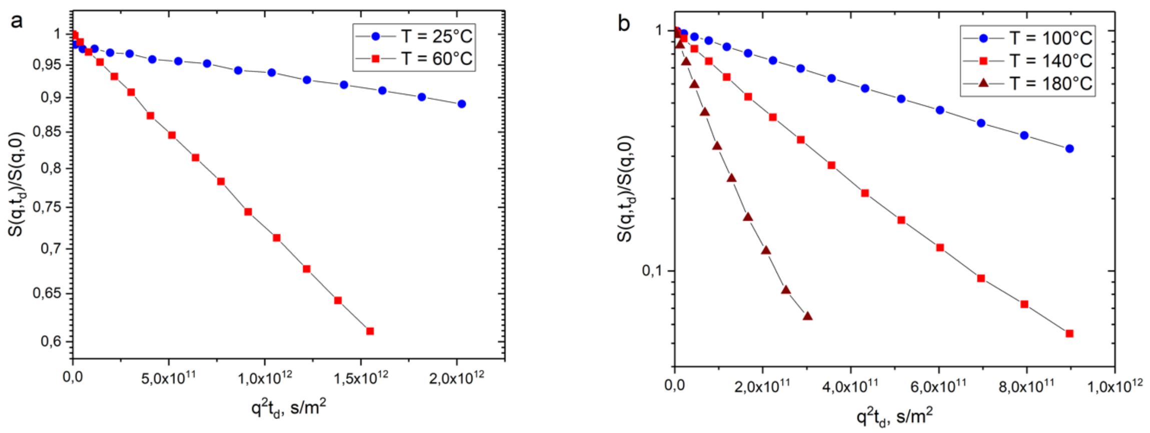

Figure S4 Spin-echo diffusion attenuation function measured for the sample PBO12 at the temperatures 25, 60, 100, 140 and 180ºC.

References

(1) Allgaier, J.; Willbold, S.; Taihyun, C. Synthesis of Hydrophobic Poly(Alkylene Oxide)s and Amphiphilic Poly(Alkylene Oxide) Block Copolymers. *Macromolecules* **2007**, *40* (3), 518–525 DOI: 10.1021/ma062417g.

(2) Gerstl, C.; Schneider, G. J.; Pyckhout-Hintzen, W.; Allgaier, J.; Willbold, S.; Hofmann, D.; Disko, U.; Frielinghaus, H.; Richter, D. Chain Conformation of Poly(Alkylene Oxide)s Studied by Small-Angle Neutron Scattering. *Macromolecules* **2011**, *44* (15), 6077–6084 DOI: 10.1021/ma201288a.

(3) Monkenbusch, M.; Kruteva, M.; Richter, D. Dynamic Structure Factors of Polymer Melts as Observed by Neutron Spin Echo: Direct Comparison and Reevaluation. *J. Chem. Phys.* **2023**, *159* (3), 034902 DOI: 10.1063/5.0150811.

(4) De Gennes, P. G. Coherent Scattering by One Reptating Chain. *J. Phys.* **1981**, *42* (5), 735–740 DOI: 10.1051/jphys:01981004205073500.

(5) Sharma, A.; Kruteva, M.; Allgaier, J.; Hoffmann, I.; Falus, P.; Monkenbusch, M.; Richter, D. Chain Confinement and Anomalous Diffusion in the Cross over Regime between Rouse and Reptation. *ACS Macro Lett.* **2022**, 1343–1348 DOI:

10.1021/ACSMACROLETT.2C00608.
